# Prohibited, but still present: local and traditional knowledge about the practice and impact of forest grazing by domestic livestock in Hungary

**DOI:** 10.1186/s13002-020-00397-x

**Published:** 2020-09-10

**Authors:** Anna Varga, László Demeter, Viktor Ulicsni, Kinga Öllerer, Marianna Biró, Dániel Babai, Zsolt Molnár

**Affiliations:** 1 Centre for Ecological Research, Institute of Ecology and Botany, Alkotmány út 2–4, Vácrátót, 2163 Hungary; 2 Rachel Carson Center for Environment and Society, Ludwig-Maximilians-Universität Munich, Leopoldstraße 11A, 80802 Munich, Germany; 3 Institute of Biology Bucharest, Romanian Academy, Spl. Independenței 296, 060031 Bucharest, Romania; 4 Centre for Ecological Research, GINOP Sustainable Ecosystems Group, Klebelsberg Kunó u. 3, Tihany, 8237 Hungary; 5 Research Centre for the Humanities, Institute of Ethnology, Tóth Kálmán u. 4, Budapest, 1097 Hungary

**Keywords:** Silvopastoral systems, Local knowledge, East-Central Europe, *Quercus* spp., Invasive species, *Robinia pseudoacacia*, Illegal activity, Closed forest, Acorn feeding, Leaf fodder

## Abstract

**Background:**

Forests have been grazed for millennia. Around the world, forest grazing by livestock became a controversial management practice, gradually restricted in many countries over the past 250 years. This was also the case in most Central and Eastern European countries, including Hungary, where forest grazing was a legally prohibited activity between 1961 and 2017. Until the 2010s, ecologists and nature conservationists considered it merely as a historical form of forest use. As a result, there is little contemporary scientific information available about the impact of forest grazing on vegetation and the traditional ecological knowledge associated with it. Our aim was to explore and summarize this type of knowledge held by herders in Hungary.

**Methods:**

We interviewed 58 knowledgeable herders and participated in forest grazing activities in 43 study locations across the country. The results were analysed qualitatively.

**Results:**

We revealed a living ecological knowledge tradition and practice of forest grazing in native and non-native forest stands. The impact of livestock grazing on native and non-native forests is not considerably different, in the view of the herders. For both forest types, the greatest impact of grazing was the suppression of the shrub layer, while grazing also increased the dominance and palatability (“tameness”) of the grasses. Livestock could cause significant damage to seedlings during forest grazing, but if done with care, grazing could also be an integral part of forestry management.

**Conclusions:**

Sustainability of current forest grazing practices depends on the depth of local and traditional knowledge applied and herders’ stewardship. We stress the importance of collaborating with holders of local and traditional knowledge in order to gain a better understanding of the effects of livestock grazing on vegetation in temperate forests.

## Background

In the last century, local and traditional ecological knowledge (TEK) has become threatened worldwide [1–3]. There are numerous factors behind this, such as the degradation of the natural environment and changes in traditional lifestyles [4, 5]. These have been induced and/or reinforced by centralised, top-down regulations, which have obstructed traditional human-nature connections by imposing restrictions on usage practices, and withdrawing land-use rights that keep TEK alive [6–9]. These regulations often threaten the maintenance of traditional landscape features and habitats [10]. Continuing fortress-type conservation management further exacerbates these difficulties [9].

Despite these circumstances, pastoral communities possess living traditional ecological knowledge that may offer solutions for resilient natural resource management around the world [9]. To maintain and support these activities at a time of dramatic change in socio-ecological systems, it requires cooperation between different stakeholder groups, for example, by carrying out knowledge co-production [8, 11–13].

Grazing based on traditional knowledge aims to adapt grazing practices to local ecological conditions while striving for long-term sustainable use of the natural resources in the given landscape [14, 15]. Traditional extensive grazing, for example, depends on landscape-scale mobility between areas with different ecological attributes. In many landscapes, this includes practices of grazing in closed-canopy forests [7, 9, 12].

Worldwide, traditional grazing began to be repressed in the 18–19th century, during the Enlightenment and colonization movements, when countless commons were enclosed, animal husbandry was intensified, and forest grazing was limited [16–18]. In consequence, pastoral communities were transformed, and grazing-related traditional ecological knowledge began to transform, as well [16, 18–20]. Recent recognition, however, points to the fundamental role of pastoral communities in sustaining ecosystem services throughout their landscape-wide and extensive grazing activities, for example, healthy and safe food production, climate change mitigation, and the maintenance of cultural values [7, 21, 22]. Despite the increasing recognition, forest grazing is still restricted in many countries worldwide. These processes have led to the emergence of conflicts between the different stakeholder groups (hunters, forestry, local communities, and nature conservation) interested in the use of forest products [13, 23–25]. For example, many foresters argue that grazing destroys forest soil and seedlings [23, 25]; hunters reported that forest grazing disturbs the activity of game and hunting ([23], Varga et al. unpublished); nature conservation often blames forest grazing for destroying natural communities [26]. At the same time, top-down, fortress-type conservation deprives local communities from grazing areas in forests [27].

Forest grazing started to be acknowledged mainly by environmental historians, ethnobiologists, nature conservationists, and community-based conservation projects. These projects focus on grazed forests as an essential grazing habitat type and living area within mobile grazing systems, and on the pastoral communities sustaining them [25, 27, 28]. This process was empowered recently in Europe by the agroforestry innovation movement [29]. Across Europe, forest grazing by livestock became increasingly referred to as a historical land-use practice [16, 18, 23]. In the last decade, however, the socio-ecological and economic importance of silvopastoral systems has gained growing recognition all over Europe [12, 20, 30]. A recent review [25] pointed out that while it would be important to know more about livestock forest grazing for conservation and management purposes, there is relatively little research available, constituting a substantial knowledge gap. Öllerer and colleagues [25] suggest that in many cases, this knowledge gap could be filled by documenting forest grazing-related local and traditional ecological knowledge. Such documentation would provide information about the ecological impacts of grazing and facilitate the innovative adaptation of forest grazing practices. Multiple ecologists acknowledge that medium-intensity grazing of forests may contribute significantly to sustainable and profitable land-use, for example, via biodiversity maintenance or food security, and may also help prevent forest fires [30, 31].

Only a few research projects in temperate regions, most of which were conducted in Asia, deal with existing local and traditional ecological knowledge about forest grazing [32, 33]. In Europe, publications on local and traditional knowledge have mostly been conducted in the boreal region, focused on forest grazing of reindeer herds, and the effects of this practice [34]. In the temperate region of Europe, data are available in the grey literature (e.g. [35]), historical ethnographic studies (e.g. [28]), and historical ecological studies (e.g. [36, 37]). At the same time, in the course of our research into traditional ecological knowledge in East-Central Europe, we have met many herders who regularly practise forest grazing, despite 200 years of restrictions and more than 50 years of outright prohibition [12].

Our main research questions were as follows:

- Do Hungarian herders possess detailed traditional ecological knowledge about the impact of forest grazing on vegetation?
- On what aspects of forest grazing do herders have knowledge and how do they utilize it during forest grazing?

The general aim of our study is to document the TEK related to forest grazing and the reported impacts of grazing on forest vegetation. We also discuss how TEK complements prevailing scientific knowledge.

In this paper, forest was defined according to the Hungarian Forestry Law [38] as “*a piece of land (min. 0.5 ha) where tree canopy cover is at least 30%*”. The term includes native and non-native forests and plantations. We focused on native and non-native closed canopy forests, where the total cover of the canopy is more than 60%. Collecting data about wood pastures was outside the scope of this study. We define forest grazing as any livestock activity in closed canopy forests, including feeding, resting, or crossing over under human control [25].

## Material and methods

### Study area

Our research was conducted in Hungary (Carpathian Basin, East-Central Europe). In this region, the climate is subcontinental; the potential natural vegetation is dominated by oak, hornbeam, and beech forests in hills and mountains, and forest-steppe forests, dry loess, and sand grasslands, and riparian vegetation in the lowlands [39].

Forests cover slightly more than 20% of the total territory of Hungary. Nearly 60% of the total forest area is made up of native tree species (mainly oaks—*Quercus robur*, *Q. petraea*, *Q. cerris*, beech—*Fagus sylvatica*, and hornbeam—*Carpinus betulus*), while the share of forest dominated by non-native tree species (black locust—*Robinia pseudoacacia*, poplar—*Populus* spp.) is about 40%. Eighty-five percent of forests are less than 80 years old and are managed mainly by rotation forestry. About 60% of forests are state-owned [40]. Forest management under all types of ownership is strictly regulated by the forest law and supervised by state authorities [41].

Based on our previous research, the forest is used for grazing all year round even nowadays, but it plays a particularly important role in late winter and early spring, as early edible grasses grow primarily in black locust forests. In summer, the forest can provide shelter from hot and stormy weather. During autumn and snow-free winters, wild fruits and acorns are important complementary feeds for any livestock. Closed canopy areas are widely used as resting places during the warmer part of the day, around noon, and the early afternoon [12].

Forest grazing in this region was practised in accordance with strict communal rules for centuries. Hungary began to impose restrictions from the 18th century onwards, as the country engaged with the Enlightenment and undertook a German-style of forestry management [24]. Later, following the introduction of the strictest regulations, the practice of forest grazing was prohibited in 1961, and breaches were routinely punished [42]. This changed significantly in 2017, when the new forestry act permitted forest grazing with sheep and cattle in forests dominated by non-native tree species. The prohibition still stands in most native forests. In Natura 2000 areas, the practice is only permitted under the supervision of the relevant authorities [40]. As the legal amendment and its method of application are recent, we did not have the opportunity to study authorised forest grazing for this publication. The livestock most frequently grazed in forests in Hungary today are sheep and cattle [12].

Forest grazing data were collected about native forests: native oak forests (in 25% of interviews), mixed forests dominated by native tree species (14%), beech forests (6%), and native pine forests (2%). Further interviews addressed grazing in non-native forests: black locust forests (26%), mixed forests dominated by non-native tree species (17%), non-native pine forests (4%), and hybrid poplar stands (7%). The studied forests are typically maintained under a rotational forest management system.

### Data collection

We interviewed 58 knowledgeable herders in 43 study locations around Hungary between 2017 and 2019. All of them are or used to be engaged in grazing, and their grazing practice also involved and/or still involves forests (Table 1). We conducted semi-structured interviews [43], which typically lasted 90–200 min. The questions dealt with the effects of grazing on the different forest layers, from the soil to the canopy; how to practise forest grazing; the types of difficulties and conflicts that may arise from grazing, and ways of preventing or solving them. During our research, we also carried out participant observations at 70% of the study locations, to better understand forest grazing practices and to triangulate interview findings with on-site field observation. In total, we spent 80 days in the field between 2017 and 2019. One of the major difficulties of our research was that we were interviewing people about a prohibited activity, so our interviewees were chosen by an acquaintance or personal recommendation, and the interviews were always preceded by extensive, less formal conversations. Although the sample size may seem small, considering the illegal status of forest grazing, until recently, and the ongoing lack of awareness concerning the termination of the prohibition in many places, we managed to collect sufficient data from most landscape types in the country. Saturation of information indicated that the sample was representative. With the permission of the informants, we recorded the interviews, and also made extensive hand-written notes. Due to the prohibited nature of the activity, we guaranteed the informants’ full anonymity. During our research, we adhered to the code of ethics of the International Society of Ethnobiology [44] and the GDPR of the European Union [45].

**Table 1:** Data about the herders and their connection with forest grazing

	***N*** = 58	100%
Male	46	79%
Female	12	21%
Under 35 years of age (the youngest was 21)	1	2%
35–65 years of age	34	58%
Over 65 years of age (the oldest was 94)	23	40%
Highest level of education: primary	24	41%
Highest level of education: secondary	22	38%
Highest level of education: tertiary or graduate level	11	19%
Level of education unknown	1	2%
Has knowledge from the period before 1961	38	67%
Has knowledge from the period 1961–2017	50	87%
Knowledge also acquired from a family member	40	69%
Knowledge also acquired from older herders	32	55%
Has practised forest grazing before 2017	58	100%
Has practised forest grazing between 2017 and 2019	36	64%
Has practised forest grazing in her/his own forest	20	34%
Has practised forest grazing in a forest owned by others	38	66%
Grazes or has grazed sheep	45	78%
Grazes or has grazed cattle	22	38%

### Data analysis and interpretations

The findings were processed and analysed qualitatively [43]. As a first step, the data from the transcribed interviews and questionnaires were interrogated using content analysis to recognize common topics and subjects. After that, we developed a coding system based on indicators of forest grazing impact structured according to forest vegetation layers (soil, herb layer, shrub layer, canopy layer) [25] and the main topics and phenomena mentioned by the herders. As a next step, we built up a database of the coded text and counted the numbers of mentions (with substantial information on forest grazing) of each indicator for the two different livestock species, sheep and cattle. In every case, we specified whether the text related to a native (NAT) or non-native forest (NonNAT), or a mixture of native and non-native stands (NAT and NonNAT). The last category covers three situations (a) data on mixed stands, (b) situations when herders did not differentiate between the two main forest types, and (c) where the reported situation is valid for both native and non-native forest types. The data were classified into four groups according to the number of mentions, as a proxy of knowledge embeddedness: a few mentions (less than 10), many mentions (10–40), mentioned by most informants (40–50), mentioned by almost all informants (50+). Participatory observations were used to interpret the material of the interviews. In the “Results” section below, we present some of the most typical and particularly interesting responses in the form of translations of verbatim quotes, indicated with italicized text. Direct quotes are differentiated by forest cover type where applicable; where the included quote refers to both vegetation types, we have not mentioned either.

## Results

### Living forest grazing practices

**Fig. 1:**
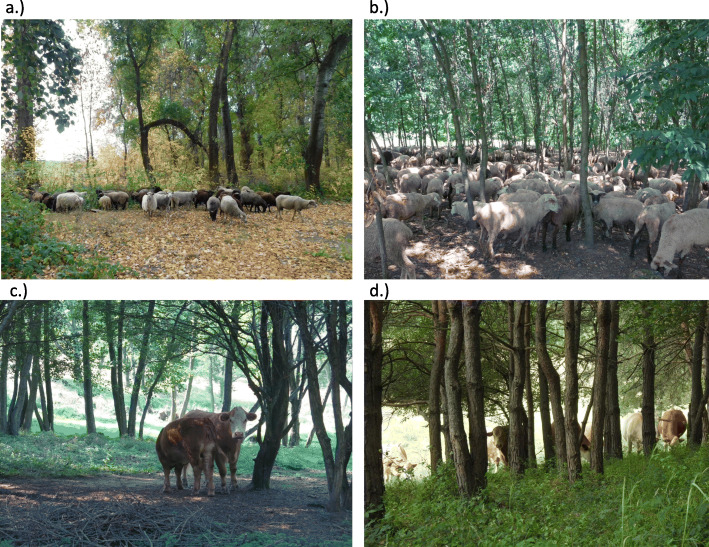
Forest grazing in native and non-native forest stands in Hungary. **a** Sheep grazing in a native poplar forest. “*They calm down and get used to the forest. If an animal has never seen* [a forest] *before, it is very hard to drive it in. You can make them get used to it, but you have to keep them at the front, because they know what is inside* [and that is why they run].” (NonNAT, NAT); **b** Sheep resting at midday in a non-native black locust stand. “*It was trimmed down so much that you can see a long way into the black locust forest… Because the sheep keep it so tidy. Even a gardener couldn’t keep things tidier*.” (NonNAT); **c** Cattle grazing in a native mixed forest. “*The time can be determined, but not by me saying two weeks or 12 days, but when you go through the forest, driving the livestock, you can see ‘this is the last time we come here’, or you wait for the next heavy rain, and then two more weeks, so that everything can freshen up again a little*.” (NAT); **d** Cattle grazing in a pine stand. “*You can see* [the effect] *on the herb layer, because it makes the grass greener and more beautiful again. It affects the undergrowth, but not the trees. In the places where we usually go inside* [the forest], *there are no shrubs, but further in, where we don’t go, there is already a dense shrub layer.*” (NonNAT, NAT) (photos: **a**: Mihály Makkai, **b**, **c**: Anna Varga, **d**: Viktor Ulicsni)

Herders reported carrying out forest grazing in native, non-native, and mixed native and non-native forests (Fig. 1). Data on sheep grazing in the forest refer mainly to non-native forest stands (50%), followed by native (33%) and native and non-native mixed stands (17%). Data on forest grazing of cattle were also obtained for the most part in native forest stands (55%), as well as in non-native (27%), and native and non-native mixed stands (18%).

Most herders stated that forest grazing was generally good for livestock, although in many cases forests were only used as supplementary pastures (in most cases it accounted for a maximum of 10–20% of total grazing time, but in some cases, forest grazing represented up to 50% of the activity). They said, for example, that grass began to grow earlier in the forest, that forest grass was “*more tender and sweeter*”, that there were hardly any gadflies and livestock could also use the shrubs to scratch off insects. It was cool and shady, protected from wind and rain, and when the grass on the open pasture dried out in the forest, it was still green. “*They could eat a variety of nutrients*” because “*more vitamin-plants grew*”. Forest grazing was sometimes used as a last resort, as “*cattle could not eat enough from forest grazing alone*”.

### Local and traditional knowledge about the impacts of forest grazing on vegetation

**Fig. 2:**
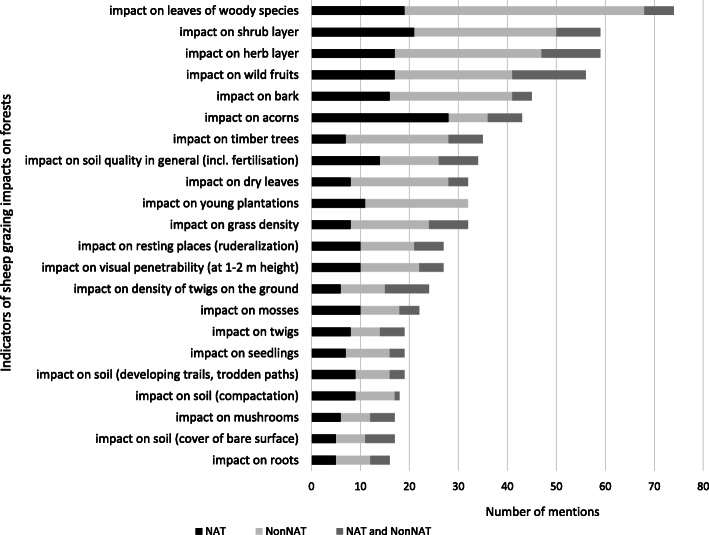
Frequency of reported indicators of forest grazing impacts according to sheep, by forest type (NAT—native, NonNAT—non-native, NAT and NonNAT—not specified)

**Fig. 3:**
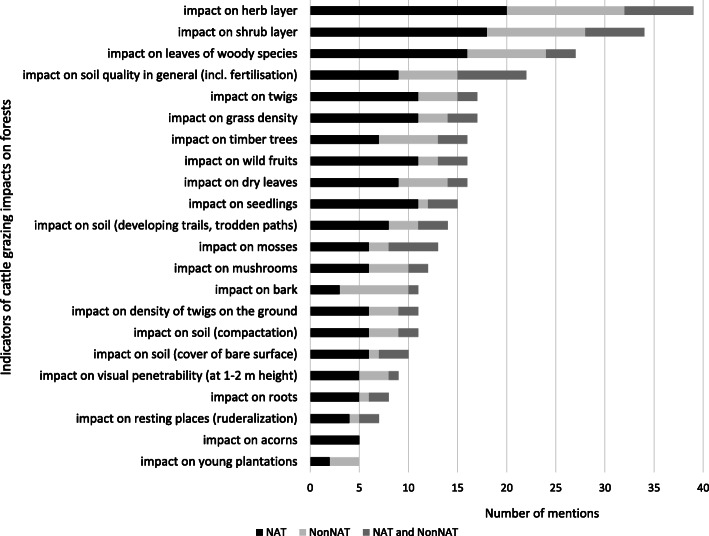
Frequency of reported indicators of forest grazing impacts by cattle, according to forest type (NAT—native, NonNAT—non-native, NAT and NonNAT—not specified)

We collected substantial information about 22 forest indicators (Figs. 2 and 3). Most herders mentioned that herb and shrub layers (especially shoots and green leaves) were impacted by grazing. This was the case for both cattle and sheep. There were only slight differences between NAT and NonNAT forests in the pattern of forest indicators impacted by both cattle and sheep grazing.

Almost all respondents agreed that the general effect of grazing in both native and non-native stands was that the forests became more penetrable, often using the word “*clearer*” (Figs. 1, 2, and 3). “*It was good because the sheep ate well and the forest became nice and clear* [visually and physically penetrable understory layer]. *It was a joy to see how tidy it all was. They tidied up the whole forest*.” (NonNAT).

With regard to the impact of livestock on forest soil, most herders mentioned trampling and fertilising. The type and degree of impact could be influenced by a number of factors. Most herders said that trampling broke up the litter and smaller branches and twigs, and therefore accelerated their decomposition, while on the other hand, it could also cause soil compaction, although only on regularly used trails and resting grounds (Figs 2 and 3). “*The amount* [of woody debris] *was not reduced, but it did get broken up, because it was worked into the ground. They break it, it snaps and snaps again, from all their little feet. It is common in alder stands, that’s the spectacular one, because they break easily*.” (NAT)

In speaking about trampling, many herders distinguished between sandy and heavier soils, but they also took into account the tree species composition of the forest; the varying amount of litter produced by different tree species affected the impact of trampling. A few informants said that, due to the thick layer of litter in oak stands, there was not a noticeably significant effect of trampling. In resting grounds, almost all informants mentioned excessive trampling, and the impact of intensive fertilising, the latter having the capacity to burn out the vegetation and stimulate nitrophilous weed growth (e.g. *Urtica*). Almost all respondents said that trails were developed where the livestock moved regularly: “*You could see trails wherever* [the livestock] *were driven into the forest in the same place. But once they were in the forest, the animals spread out in all directions, and the whole area was the same*.” (Figs. 2 and 3).

Many herders regarded fertilising as positive for the soil. A few noted that when the stock of animals was low and spread out, the impact of fertilising was barely noticeable.

#### Ground layer vegetation

All interviewees emphasised that neither sheep nor cattle had any significant impact on roots. Only a few mentioned that the sheep sometimes chewed roots protruding from the soil (Figs. 2 and 3). Many herders reported that neither cattle nor sheep had any significant impact on the moss layer, which is mainly found in native forests. Sheep sometimes nibbled the moss, some respondents noted. A few herders mentioned the impact of trampling, causing dry cushions of moss to be broken to pieces (Figs. 2 and 3).

The most noticeable effect of forest grazing on the herb layer was caused during resting. When sheep and cattle regularly rested in the same place, the herb layer disappeared in both native and non-native forests. When these areas were no longer used for resting livestock, weeds proliferated.

Almost all respondents were unanimous in their opinion that in both types of forest, grazing (chewing, trampling, and fertilising) resulted in the herb layer becoming “*tamer*” (more palatable, thus more suitable for grazing): grasses flourished (e.g. *Bromus sterilis* and *Elymus repens*, but also *Stellaria media* in NonNAT forests), grass tussocks became bushier, and certain weeds (e.g. *Urtica dioica*, *Rubus* spp.) were suppressed (Figs. 1, 2, and 3). “*When it is grazed continuously, every year, the forest floor will be even grassier. If it is not grazed, nettles and bramble shoots will take over the forests sooner than grass. But if it is grazed constantly, it will be covered in grass sooner or later*” (NonNAT). Only a few herders mentioned rare or specialist herbaceous species (e.g. *Dictamnus albus, Pulmonaria officinalis, Urtica kioviensis*).

#### Regeneration and shrub layer, young forests

The interviewed herders were nearly unanimous in stating that in both native and non-native forests, grazing of the shrub layer was secondary or supplementary in character, but that it reduced the amount of shrub cover considerably. According to most herders, the sheep impacted this layer mainly by grazing leaves and the ends of shoots (Fig. 2). The impact of cattle was mostly by trampling (with many herders also mentioning the effect of cattle rubbing against the shrubs), although older shrubs were generally less affected. “*If* [the sheep] *have other things *[to eat], *they don’t really eat the shrubs*.” The decrease in shrub cover made the forest even more grazable, or “*tamer*”, as most herders put it. “[Shrubs] *die back when the livestock keep coming and nibbling. They grow high at the top, like a Christmas tree, as their core grows further up, because* [the livestock] *can’t reach it*.”

Many herders said that the impact on saplings and young trees was determined by the type of forest management. Seedlings that emerge from the forest floor have their leaves and shoots eaten by sheep and cattle. “*The new growth got snapped up*.” In young forests, grazing was generally prohibited for 3–7 (12) years, until the shrubs had “*grown past the mouths of the livestock*” (approx. 1.5–2 m). In younger forests, most informants stated that grazing hindered the growth of saplings, causing deformities in their shape (bushy growth), while trampling, rubbing against them, and bark removal could even destroy these smaller trees. “*Sometimes the sheep went into a young forest. They chewed off the tops, and although* [the sapling] *did not dry out, it did not grow so radiant, and actually became bushy and full of branches, which is not good for it. The main shoot goes further, they can’t bite that off, and they can graze* [in the forest]; *if they chew off the lower branches, that doesn’t cause harm*.” (NonNAT). A few herders emphasised that in NonNAT coppice forests especially, careful grazing could be beneficial to the growth of saplings (e.g. black locust) by removing the weeds and side branches, by chewing away the less valuable sucker shoots (by the time the root shoots appear, sheep are driven away from the area).

A few herders mentioned conscious interventions to improve forest grazing areas. “*We usually cut down the forest edges, where hawthorn and blackthorn grow bushy, so that the livestock can get into the forest*” (NonNAT); and “*We also cut down the thorny bushes that can be found in the forest*” (NAT).

#### Tree leaves, wild fruits, acorns

Almost all respondents said that the livestock ate not only the leaves of saplings but also the accessible green leaves of the canopy (a sign of this was that the bottom of the canopy appeared to have been cut straight off, at a height of 1.5–2.5 m, depending on the height of the livestock) (Figs 1, 2, and 3) “*When* [the branches] *bent over because of the rain or stormy weather*, [the sheep] *would go for them*” (NonNAT). A few herders said that, when food was scarce, using a hooked stick, they would pull down the leafy branches of oak, hazel, ash, and black locust trees from a height of up to 4 (or even 8) m. In most cases, the livestock also happily ate fallen, frostbitten leaves (“*we were happy when a storm came*”).

Likewise, most herders spoke about the sheep and cattle eating wild fruit (Figs. 2 and 3): “*They eat cherry plum, they like it. I sometimes knock it down for them*.” Many herders emphasised that the livestock knew which wild pear trees bore sweet fruit and “*hurried towards them*”.

Where possible, an important part of forest grazing practice for both sheep and cattle was mast feeding in oak and beech stands: “*It improves the sheep*”. Many informants reported that when there were few acorns, the livestock would rush from one acorn-bearing tree to the next, and they would easily grow bored. A few herders said that it was not the amount of mast-consumption that drove the development of vegetation, but the weed grazing that accompanied eating acorns, that is, the process of keeping the grass layer “*clear*”.

#### Trees and stand structure

“*In the forest, the trees stretch upwards. If they* [the livestock] *graze the saplings down, it gets thinned out, but that changes nothing on the older trees*.” (NonNAT, NAT). Almost all informants agreed that forest grazing over a period of decades had a minimal impact on canopy closure and on the integrity of tree stands. Most herders held the view that the canopy layer was affected more by forest management and by ecological conditions than by grazing (Figs. 2 and 3). “*In truth, the herd can’t do as much damage* [in the forest] *as if it were cut down, that’s obvious*.”

Most herders reported trees generally benefited when the forest floor was clear and the soil was fertilised. “*It’s better for the forest as well to have enough air*,” and “*shrubs take away moisture* [from the trees]” (NonNAT, NAT).

A few herders mentioned that damaging tree bark is a negative effect of grazing (cattle rubbing against it, sheep and cattle chewing the bark of ash and young black locust trees) (Fig. 3). One herder said that black locust bark “*is bitter, and it does for stomach problems what Unicum* [a famous traditional Hungarian bitter] *does for people*”. A few emphasised that in medium-age forests, livestock would most typically chew bark when they were “*bored*” or salt-deficient. Only a few herders talked about collecting standing deadwood: “*With a hooked stick we pulled down the dead branches from a height of 5-6 metres, for firewood*.”

### Herders’ forest grazing practices

**Fig. 4:**
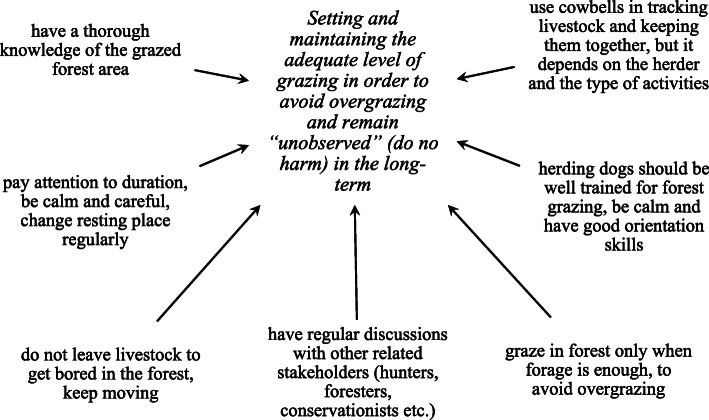
Local and traditional knowledge-based rules for careful and sustainable forest grazing practice, as mentioned by the herders

In the forest, herders stated, greater care needed to be taken of the livestock, as they were more sensitive than in the open: thorns could injure their udders or their hooves—even the dog had to be careful. A few herders stated that the livestock was calmer and did not run so much as on grasslands (Fig. 4). The general view was that the herder should take care that livestock went where they could graze well, did not get bloated, did not go into forbidden areas or cause damage (especially in neighbouring arable land, alfalfa fields, or young forests), and did not stop moving or get lost. Many herders mentioned the role of cowbells in tracking livestock and keeping them together, irrespective of forest type: “*Put it on the one about whom you are sure where it will go, the one who knows* [the forest]”. In many cases, however, cowbells were removed so passers-by could not hear that there was any livestock in the forest.

Most herders stated that the main criteria for long-term, sustainable forest grazing, in both native and non-native forests, were to avoid overgrazing and to remain unnoticed because of the legal prohibition. “*It’s hard to make up good rules. Basically, you have to keep an eye on the livestock, and that’s it.” / “If everyone did things using simple common sense, there would be no need for these rules*.”

Remaining unnoticed meant leaving no trace of grazing activity for others to see, and not allowing others to realise that the herders were breaking the law. Most herders emphasised that overgrazing was not in the herders’ interests for their livestock, either: “*then you can’t give the sheep a good feed; I am not my own enemy*”.

Livestock sometimes entered the *forest* happily (“*the sheep go crazy about* [the forest] *and* [willingly] *enter it*”), but not always (“*they needed to be enticed*”). Livestock needed to be acclimatised to being in the forest (Figs. 1 and 2). Almost all respondents agreed that it was especially important to pay attention to the method and duration of grazing and to have a thorough knowledge of the area (Fig. 4). One important basic principle was to keep moving: if the livestock stopped in the forest with a full stomach (whether daytime or night-time, but particularly in winter), they could damage the tree stand “*out of boredom*”. “*They went in, ate their fill, and came out. The old folk used to say you can take them in* [to the forest], *but keep them moving, because if you let them stop, they will start to nose around, and then they can do damage*.” (NonNAT). One herder emphasised the finer aspects of his profession, for example, the importance of the direction and speed of the herd’s movement: “*If one sheep bends a young tree over* [by crossing between two nearby trees, because of the need to change rows in plantation forests], *then the other will immediately nibble off its top*,” and “*Grazing should be done calmly, without the livestock being driven hard by the dog*,” because then the livestock does not change rows and thus cause less damage to young shoots.

Most herders argued that regular interaction with other stakeholders, especially with local foresters/hunters/nature conservationists, could foster the development of a forest grazing practice that does no damage and thus prevents conflicts (Fig. 4). Foresters: “*When they were going to cut down a patch that was ready for logging, they told us herders not to let the livestock in then. We could go around it, that’s what we were there for. We also had responsibilities; we weren’t just there for the sake of it*”. (NAT); Hunters: “*It depends on the hunter, but the basic rule is that I don’t go in the forest in stag rutting season. He* [the hunter] *calls me before starting the deer hunt, but there is one guy who does not. The deer grows so used to the cattle, and even to the dogs, that they come right up to the edge of the herd to graze. Done sensibly, grazing by herders and wild game management can both work side by side*.” (NAT); Conservationist: “*The conservation authority did not oppose grazing in 2006* [when it was still forbidden by the Forestry Act]. *Moreover, they said, let them go into the forest, at least they will get rid of the tree-of-heaven* [Ailanthus altissima].” (NonNAT).

## Discussion

### The knowledge still exists

Our research proved that this traditional form of land-use and the related knowledge still exists in Hungary today, even though the practice was prohibited for more than 50 years and incrementally restricted for the 200 years prior to that. There were not only memories of the historical impact of forest grazing on vegetation and the sustainability of forest grazing but also a majority of herders also still possessed living local and traditional ecological knowledge. Herders’ knowledge extends to different types of forests and livestock species. This is all the more surprising because Hungary is one of the countries in Europe where forest grazing has been most strictly and most severely restricted since the end of the 18th century, and where the area covered by forest has decreased most dramatically [28, 46]. The practice and the associated knowledge managed to survive thanks to several factors, such as the strength of environmental and climatic constraints (need for forest forage), and the demands of extensively grazed livestock breeds [12]. Forest grazing follows the pattern seen in the revival of other traditional activities. In addition to traditional ecological knowledge, there is a significant amount of local knowledge about forest grazing. This is especially notable among herders who come from non-herder families, or who just recently started to graze in newly established non-native forest stands [42].

Forest grazing previously occurred predominantly in oak and beech stands and floodplain forests [12, 42], whereas nowadays forest grazing is mostly practised in non-native black locust forests [47]. In beech and pine forests, there was often insufficient forage, and in poplar stands the litter layer was too thick, while black locust forests provided plenty of fresh grass at the end of winter and in early spring ([2], Demeter et al., unpubl.).

### The impact of grazing on forest vegetation

For several decades, one of the greatest challenges forest grazing faced was the fact that it was illegal. The illegality of the practice may, however, have played a part in herders learning how to graze their livestock “*unobserved*”, meaning with no considerable impact on woody species. Herders reported that going “*unobserved*” was also good for local cooperation. One reason for this is that tolerant foresters and inspectors also avoided punishment from their own superiors. On the other hand, during “*unobserved*” grazing, the herder paid close attention to not causing significant damage, which could harm and/or threaten the husbandry of the forest stand.

In the opinion of the herders interviewed, whether forest grazing exerted a beneficial or damaging impact on the forest depended to a great extent on the herder. Based on herders’ knowledge, the impact of cattle and sheep grazing on the forest vegetation is different. For example, herders more often mentioned the consumption of young woody vegetation by sheep, while they more frequently mentioned trampling damage by cattle. However, the slight differences between native and non-native and mixed forest stands were influenced not only by their vegetation but also by the fact that sheep grazing was more common in non-native and mixed forests, while cattle grazed more often in native forests.

During the interviews, herders mainly mentioned general changes in vegetation structure, with fewer mentions of the grazing on particular species. They spoke only in the most general terms about disturbance and succession and hardly mentioned grazing preferences (forage species) at all. In many instances, however, what herders said about the impact of grazing on vegetation was similar to the information documented in the ecological literature on temperate forests [25]. Examples include the graminification of the forest; the thinning of the shrub layer; the protection of young planted saplings when grazing is practised with care, versus the browsing of saplings when grazing is practised negligently; the localised compaction of soil; and the trampling and breaking up of smaller dry branches. The herders and the literature also agreed that medium-aged and old trees were demonstrably affected by forest management, and not visibly damaged by grazing. That is, grazing does not hinder timber production [33, 48]. In the traditional forest grazing system, prohibiting grazing before and after the start of regeneration is a long-standing management element [23]. This could be effectively applied today in forests managed according to the rotational forest system. A good example of this is the practice of pig husbandry and forest management in South-European floodplain forests [7]. When kept alongside excessive populations of wild game, however, forest grazing by livestock may decrease species diversity of the herb and shrub layers. Unfortunately, separating the effects exerted on forest biodiversity by the wild game from those exerted by grazing livestock constitutes an important research gap [25, 35].

Herders were surprisingly knowledgeable about how to suppress weeds and sucker shoots in black locust plantations during forest regeneration without causing damage (cf. [49]). In most instances, this activity was carried out at the request of the forester or forest owner. The possibilities for the cooperation of this kind between the grazing livestock, the herder, and forest management are discussed by Fraser and colleagues [50].

In herders’ general experience, grazed forests are clear, tidy*,* and visually penetrable. From their answers, it became clear that reducing the shrub layer and increasing penetrability may also be useful for keeping livestock together. Herders typically preferred penetrable forest stands; their opinions depended mainly on the stands’ opportunities for use (e.g. firewood, grazing). By contrast, many features regarded as positive from an ecological viewpoint (e.g. decaying, fallen or standing deadwood, dense and diverse shrub layer [26, 51]) do not align with the herders’ definition of a forest in good condition.

Acorn-feeding of cattle and sheep was referred to as a significant economic value in some drier oak and beech forests, although the phenomenon is nowhere near as widespread as it used to be [7, 42]. Ecologists of the region are generally only aware of the existence of mast-feeding of pigs, and even this is regarded as a historical form of land-use that has now disappeared [52]. One observation was the claim among herders that mast-feeding had no perceptible impact on the forest, while the grazing that took place during foraging for acorns reduced the cover of the herb layer. This observation warns us that it is not easy to interpret the exact impact that traditional land-use practices once exerted on vegetation. Contemporary ecological studies and thorough observation of livestock grazing behaviour are needed in order to carry out a reliable ecological interpretation of historical data.

Herders’ reports of their practices confirmed the assumption made by scientists (e.g. [53, 54]) that forest grazing reduces the quantity of thin deadwood in the forest (through trampling). The thought arises, however, that the person in charge of grazing the livestock may also contribute to the reduction in deadwood by collecting dry branches for firewood [23], an activity that is not accounted for in research exclusively documenting phenomena of forest dynamics (cf. [25]).

## Conclusions and outlook

Our results show that closed canopy forests still provide a meaningful resource for livestock grazing in Hungary. Nevertheless, traditional forest grazing and the knowledge underpinning it has not been sufficiently studied by ecologists, as both are mostly regarded as long lost.

Herder’s knowledge was found to cover various aspects (e.g. sustainable management, impact on forest layers) of forest grazing. The unexpected variety and depth of local and traditional knowledge of a long-banned and largely abandoned practice indicate that it would also be worthwhile studying other, similarly lesser-known traditional land-use practices (e.g. wetland grazing, forest-based pig farming), and carrying out knowledge co-production with their practitioners [11, 55]. In this way, local and traditional knowledge could contribute to the development of more adaptive conservation and land management systems [21, 25]. For example, forest grazing has a noticeable direct effect on herb and shrub layers; this impact could be directed to control invasive species.

Our research also highlights the importance of developing processes and initiatives for the rediscovery of forest grazing and mobile herding activities in general, with the aim of promoting the understanding and preservation of biocultural values. One of the pillars for maintaining these values is to enable local communities to continue to practise traditional forms of land-use, adapting them to the rapidly changing natural and economic environments.

## Data Availability

The datasets collected and/or analysed during the current study are available from the corresponding author on reasonable request.

## References

[1] Biró É, Babai D, Bódis J, Molnár Z (2014). Lack of knowledge or loss of knowledge? Traditional ecological knowledge of population dynamics of threatened plant species in East-Central Europe. J Nat Conserv..

[2] Iniesta-Arandia I, Del Amo DG, García-Nieto AP, Pineiro C, Montes C, Martín-López B (2015). Factors influencing local ecological knowledge maintenance in Mediterranean watersheds: insights for environmental policies. Ambio.

[3] Lyver PB, Timoti P, Davis T, Tylianakis JM (2019). Biocultural hysteresis inhibits adaptation to environmental change. Trends Ecol Evol..

[4] Berkes F, Colding J, Folke C (2000). Rediscovery of traditional ecological knowledge as adaptive management. Ecol Appl..

[5] Reyes-García V, Guèze M, Luz AC, Paneque-Gálvez J, Macía MJ, Orta-Martínez M, Pino J, Rubio-Campillo X (2013). Evidence of traditional knowledge loss among a contemporary indigenous society. Evol Hum Behav..

[6] Anderson KM (2005). Tending the wild: Native American knowledge and the management of California's natural resources.

[7] Gugic G (2009). Managing sustainability in conditions of change and unpredictability—the living landscape and floodplain ecosystem of the Central Sava River basin.

[8] Biró M, Molnár Z, Babai D, Dénes A, Fehér A, Barta S, Sáfián L, Szabados K, Kiš A, Demeter L, Öllerer K (2019). Reviewing historical traditional knowledge for innovative conservation management: A re-evaluation of wetland grazing. Sci Total Environ..

[9] Mattalia G, Volpato G, Corvo P, Pieoroni A (2018). Interstitial but resilient: nomadic shepherds in Piedmont (Northwest Italy) Amidst spatial and social marginalization. Hum Ecol.

[10] Bobiec A, Podlaski R, Ortyl B, Korol M, Havryliuk S, Öllerer K, Ziobro J, Pilch K, Dychkevych V, Dudek T, Mázsa K, Varga A, Angelstam P (2019). Top-down segregated policies undermine the maintenance of traditional wooded landscapes: evidence from oaks at the European Union’s eastern border. Landsc Urban Plan..

[11] Tengö M, Brondizio ES, Elmqvist T, Malmer P, Spierenburg M (2014). Connecting diverse knowledge systems for enhanced ecosystem governance: the multiple evidence base approach. Ambio..

[12] Varga A, Molnár Z, Biró M, Demeter L, Gellény K, Miókovics E, Molnár Á, Molnár K, Ujházy N, Ulicsni V, Babai D (2016). Changing year-round habitat use of extensively grazing cattle, sheep and pigs in East-Central Europe between 1940 and 2014: Consequences for conservation and policy. Agr Ecosyst Environ..

[13] Molnár Z, Kis J, Vadász C, Papp L, Sándor I, Béres S, Sinka G, Varga A (2016). Common and conflicting objectives and practices of herders and conservation managers: the need for a conservation herder. Ecosyst Health Sustain..

[14] Molnár Z (2014). 2014. Perception and management of spatio-temporal pasture heterogeneity by Hungarian herders. Rangeland Ecol Manag..

[15] Meuret M, Provenza FD (2015). 2015. When art and science meet: integrating knowledge of French herders with science of foraging behavior. Rangeland Ecol Manag..

[16] Rotherham I (2007). The implications of perceptions and cultural knowledge loss for the management of wooded landscapes: A UK case-study. Forest Ecol Manag..

[17] Sekar T (2015). Grazing and Penning practices and their impacts. Forest Management in Tamil Nadu -A Historical Perspective.

[18] Samojlik T, Fedotova A, Kuijper DPJ (2016). Transition from traditional to modern forest management shaped the spatial extent of cattle pasturing in Białowieża Primeval Forest in the nineteenth and twentieth centuries. Ambio..

[19] Zent S (2013). Processual perspectives on traditional environmental knowledge. Understanding cultural transmission in anthropology.

[20] Hartel T, Plieninger T. European wood-pastures in transition: a social- 856 ecological approach: Routledge, Taylor and Francis Group; London, New York; 2014.

[21] Biró M, Molnár Z, Öllerer K, Lengyel A, Ulicsni V, Szabados K, Kiš A, Perić R, Demeter L, Babai D (2020). Conservation and herding co-benefit from traditional extensive wetland grazing. Agric Ecosyst Environ.

[22] Schröter M, Başak E, Christie M, Church A, Keune H, Osipova E, Oteros-Rozas E, Sievers-Glotzbach S, van Oudenhoven APE, Balvanera P, González D, Jacobs S, Molnár Z, Pascual U, Martín-López B (2020). Indicators for relational values of nature’s contributions to good quality of life: the IPBES approach for Europe and Central Asia. Ecosyst People.

[23] Vera FWM (2000). Grazing Ecology and Forest History.

[24] Johann E, Agnoletti M, Bölöni J, Erol SC, Holl K, Kusmin J, Latorre JG, Molnár Z, Rochel X, Rotherham ID, Saratsi E, Smith M, Tarang L, van Benthem M, van Laar J, Parotta JA, Trosper RL (2012). Europe. Traditional forest-related knowledge.

[25] Öllerer K, Varga A, Kirby K, Demeter L, Biró M, Bölöni J, Molnár Z (2019). Beyond the obvious impact of domestic livestock grazing on temperate forest vegetation—a global review. Biol Conserv..

[26] Sandström J, Bernes C, Junninen K, Lõhmus A, Macdonald E, Müller J, Jonsson BG (2018). Impacts of dead wood manipulation on the biodiversity of temperate and boreal forests. A systematic review. J Appl Ecol..

[27] Yılmaz E, Zogib L, Urivelarrea P, Demirbaş S (2019). Mobile pastoralism and protected areas: conflict, collaboration and connectivity. Parks.

[28] Varga A (2017). ‘Innovation from the Past.’ Silvopastoral Systems in Hungary in the Light of Hungarian Ethnographic Literature. Acta Ethnogr Hung..

[29] Damianidis C, Santiago-Freijanes JJ, den Herder M, Burgess P, Mosquera-Losada MR, Graves A, Papadopoulos A, Pisanelli A, Camilli F, Rois-Dıaz M, Palma JHN, Pantera A. Agroforestry as a sustainable land use option to reduce wildfires risk in European Mediterranean areas. Agroforestry Systems. 2020. p. 1–11. 10.1007/s10457-020-00482-w.

[30] Rois-Díaz M, Lovric N, Lovric M, Ferreiro-Domínguez N, Mosquera-Losada MR, den Herder M, Graves A, Palma JHN, Paulo JA, Pisanelli A, Smith J, Moreno G, García S, Varga A, Pantera A, Mirck J, Burgess P (2018). Farmers’ reasoning behind the uptake of agroforestry practices: evidence from multiple case-studies across Europe. Agrofor Syst..

[31] Moreno G, Aviron S, Berg S, Crous-Duran J, Franca A, de Jalón SG, Hartel T, Mirck J, Pantera A, Palma JHN, Paulo JA, Re GA, Sanna F, Thenail C, Varga A, Viaud V, Burgess PJ (2018). Agroforestry systems of high nature and cultural value in Europe: provision of commercial goods and other ecosystem services. Agrof Syst..

[32] Norbu L (2002). Grazing management in broadleaf forests—Bhutan. J Bhutan Stud..

[33] Buffum B, Gratzer G, Tenzin Y (2009). Forest grazing and natural regeneration in a late successional broadleaved community forest in Bhutan. Mt Res Dev.

[34] Roturier S, Roué M (2009). Of forest, snow and lichen: Sámi reindeer herders’ knowledge of winter pastures in northern Sweden. Forest Ecol Manag..

[35] Humphrey J, Gill R, Claridge J (1998). Grazing as a management tool in European forest ecosystems.

[36] Bürgi M, Gimmi U (2007). Three objectives of historical ecology: the case of litter collecting in Central European forests. Landscape Ecol..

[37] Bürgi M, Gimmi U, Stuber M (2013). Assessing traditional knowledge on forest uses to understand forest ecosystem dynamics. Forest Ecol Manag..

[38] The text of the Hungarian Forestry Law (LVI (26.05) Act of 2017) on forests, forest protection and forestry, as in force [in Hungarian] https://net.jogtar.hu/jogszabaly?docid=a0900037.tv Accessed 13 May 2020.

[39] Molnár Z, Király G, Fekete G, Aszalós R, Barina Z, Bartha D, Biró M, Borhidi A, Bölöni J, Czúcz B, Csiky J, Dancza I, Dobor L, Farkas E, Farkas S, Horváth F, Kevey B, Lőkös L, Magyari E, Molnár VA, Németh C, Papp B, Pinke G, Schmidt D, Schmotzer A, Solt A, Sümegi P, Szmorad F, Szurdoki E, Tiborcz V, Varga Z, Vojtkó A, Kocsis K (2018). Vegetation. National Atlas of Hungary: Natural Environment.

[40] Aujeszky P (2019). Környezeti helyzetkép, 2018.

[41] Jáger L, Schiberna E, Ali TG, Horváth K (2015). Forest land ownership change in Hungary.

[42] Varga A, Heim A, Demeter L, Molnár Z, Roué M, Molnár Z (2017). Rangers bridge the gap: integration of wood-pasture related traditional ecological knowledge into nature conservation. Knowing our Land and Resources: Indigenous and local knowledge of biodiversity and ecosystem services in Europe & Central Asia. Knowledges of Nature 9.

[43] Newing H, Eagle CM, Puri RK, Watson CW (2011). Conducting research in conservation: social science methods and practice.

[44] International Society of Ethnobiology (2006). International Society of Ethnobiology Code of Ethics (with 2008 additions).

[45] The text of the General Data Protection Regulation, regulation (EU) 2016/679 of the European Parliament and of the Council, of 27 April 2016 https://gdpr-info.eu/ Accessed 13 May 2020.

[46] Biró M, Bölöni J, Molnár Z (2018). Use of long-term data to evaluate loss and endangerment status of Natura 2000 habitats and effects of protected areas. Conserv Biol..

[47] Vítková M, Sádlo J, Rolecek J, Petrík P, Sitzia T, Müllerová J, Pyšek P (2020). Robinia pseudoacacia-dominated vegetation types of Southern Europe: Species composition, history, distribution and management. Sci Total Environ..

[48] Noack FAW, Manthey M, Ruitenbeek JH, Mohadjer MRM (2010). Separate or mixed production of timber, livestock and biodiversity in the Caspian Forest. Ecol Econ.

[49] McEvoy PM, McAdam JH (2008). Sheep grazing in young oak Quercus spp. and ash Fraxinus excelsior plantations: vegetation control, seasonality and tree damage. Agrofor Syst..

[50] Fraser EC, Kabzems R, Lieffers VJ (2001). Sheep grazing for vegetation management in the northern forests of British Columbia and Alberta. Forest Chron..

[51] Grindean R, Tanţău I, Feurdean A (2019). Linking vegetation dynamics and stability in the old-growth forests of Central Eastern Europe: Implications for forest conservation and management. Biol Conserv..

[52] Szabó P (2012). Sources and methods to reconstruct past masting patterns in European oak species. Arboricult J..

[53] McEvoy PM, Flexen M, McAdam JH (2006). The effects of livestock grazing on ground flora in broadleaf woodlands in Northern Ireland. Forest Ecol Manag..

[54] Smale MC, Dodd MB, Burns BR, Power IL (2008). Long-term impacts of grazing on indigenous forest remnants on North Island hill country. New Zealand. New Zeal J Ecol..

[55] Babai D, Molnár Z (2014). Small-scale traditional management of highly species-rich grasslands in the Carpathians. Agr Ecosyst Environ..

